# Diverse mycotoxin threats to safe food and feed cereals

**DOI:** 10.1042/EBC20220221

**Published:** 2023-09-13

**Authors:** Rosie L. Latham, Jeremy T. Boyle, Anna Barbano, William G. Loveman, Neil A. Brown

**Affiliations:** 1 Milner Centre for Evolution, Department of Life Sciences, University of Bath, Bath, U.K.; 2 Department of Life Sciences, University of Bath, Bath, U.K.

**Keywords:** Aspergillus, Cereals, Fusarium, Mycotoxins

## Abstract

Toxigenic fungi, including *Aspergillus* and *Fusarium* species, contaminate our major cereal crops with an array of harmful mycotoxins, which threaten the health of humans and farmed animals. Despite our best efforts to prevent crop diseases, or postharvest spoilage, our cereals are consistently contaminated with aflatoxins and deoxynivalenol, and while established monitoring systems effectively prevent acute exposure, *Aspergillus* and *Fusarium* mycotoxins still threaten our food security. This is through the understudied impacts of: (i) our chronic exposure to these mycotoxins, (ii) the underestimated dietary intake of masked mycotoxins, and (iii) the synergistic threat of cocontaminations by multiple mycotoxins. Mycotoxins also have profound economic consequences for cereal and farmed-animal producers, plus their associated food and feed industries, which results in higher food prices for consumers. Climate change and altering agronomic practices are predicted to exacerbate the extent and intensity of mycotoxin contaminations of cereals. Collectively, this review of the diverse threats from *Aspergillus* and *Fusarium* mycotoxins highlights the need for renewed and concerted efforts to understand, and mitigate, the increased risks they pose to our food and feed cereals.

## *Aspergillus* and *Fusarium* mycotoxins in cereals

Fungi are major crop pathogens and food spoilage moulds, which can contaminate our food and animal feed with an array of toxic secondary metabolites, referred to as mycotoxins [1]. Consumption of these mycotoxins is hazardous to humans and animals, with some mycotoxins being toxic at nanomolar concentrations [1,2]. Approximately 300–400 mycotoxins have been identified, where their consumption has been associated with cancers, neurological issues, and immune suppression [1]. Two prominent genera of mycotoxigenic fungi, *Aspergillus* and *Fusarium*, are responsible for the contamination of our major crops [3]. At present, mycotoxins produced by these genera are regarded as toxins of great importance and include aflatoxins, deoxynivalenol (DON), fumonisins, T-2 and HT-2 toxins, and zearalenone [4]. The opportunistic pathogens and food spoilage moulds, *Aspergillus flavus* and *Aspergillus parasiticus*, can both contaminate cereals (particularly maize) in the field and during storage, where they are known to produce aflatoxins, which are highly toxic and are classified as class I human carcinogens by the International Agency for Research on Cancer, in addition to causing digestive problems, plus reproductive and fertility issues [5–9]. Fusarium Head Blight is a key floral disease of cereals, primarily caused by *Fusarium graminearum*, a top ten fungal pathogen that produces mycotoxins such as DON and zearalenone, which reduces crop yields and grain quality, while posing a health risk [10–13]. The greatest risk of exposure to humans and farmed animals is through the consumption of mycotoxin-contaminated food or feed, which causes significant safety complications throughout our existing food and feed supply chains.

Of the global crop supply, an estimated 60–80% of crops are contaminated by mycotoxins, with 20% of crops exceeding the European Union (EU) legal food safety limits [14]. Cereals are a staple food, accounting for a global cropping area of 700 million hectares, which supplies approximately 40% of the energy and protein intake in a human diet [15]. Major cereal crops used for food and animal feed are particularly susceptible to mycotoxin contamination. In addition to threatening human health and food safety, mycotoxins cause significant economic costs, through their associated impacts on crop yields, animal productivity, and international trade [16].

Given the impacts on human and animal health, governments, such as the EU commission, set legal limits on permitted mycotoxin levels for food and depending on the specific mycotoxin, higher legal limits, or only recommendations, are set for animal feed [17]. Often, when mycotoxin levels are too great to be used for food, the cereal grains can be downgraded to animal feed; however, this comes at an economic cost to the cereal producer [18]. For example, 2 µg/kg aflatoxin and 750 µg/kg DON are permitted in unprocessed cereals for direct human consumptions, but 20 µg/kg aflatoxin and 8000 µg/kg DON are allowed in animal feed [19,20]. Both *Aspergillus* and *Fusarium* species can infect cereal crops in the field, inhibiting crop development, affecting grain production, and resulting in yield losses [8,13,21,22]. Aflatoxins contamination of crop seeds impacts crop growth and results in loss of market value [22]. *Fusarium* mycotoxins include DON, zearalenone, and fumonisins, can inhibit plant development by interfering with various metabolic processes. For example, DON has a negative impact on the growth of wheat seedlings [23].

To mitigate mycotoxins within our food and feed supply chains, governmental organisations such as the European Food Safety Agency (EFSA) and agribusinesses, such as (DSM BIOMIN, World Mycotoxin Survey) have established networks to monitor mycotoxin occurrence in food and animal feed [14,24]. Whilst these limits are effective at preventing acute human exposure to mycotoxins, the impact of *Aspergillus* and *Fusarium* mycotoxins still threatens farmed animal health, our economic sustainability, and the security of maintaining a safe supply of food and feed cereals. Understanding the different natures and diverse impacts of these hazardous fungal contaminants of cereals is vital in assessing how the associated mycotoxin threats may change in our future environments.

## Multi-level economic and health impacts

**Figure 1:**
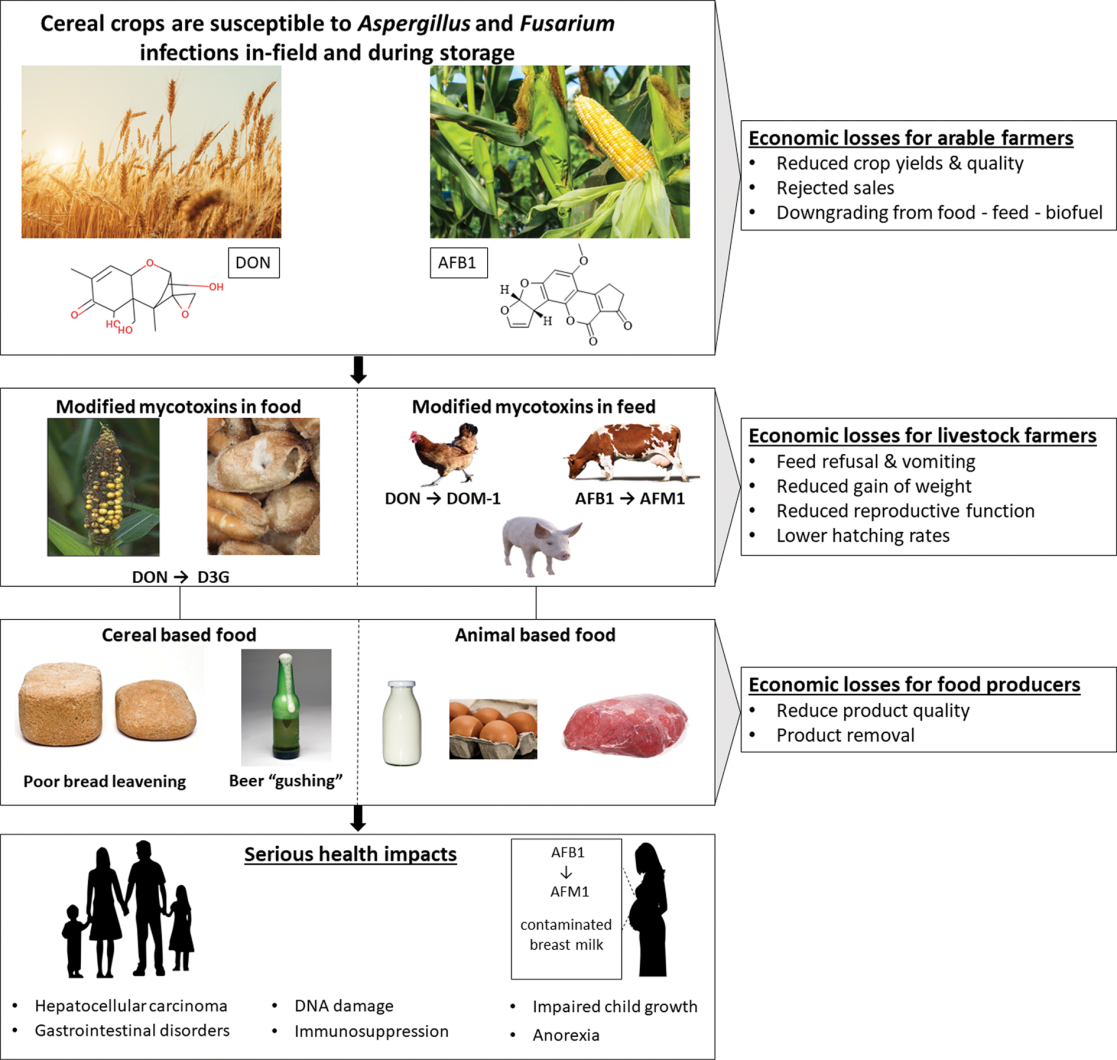
Economic and health impacts of mycotoxin in the food and feed supply chains Highlights the impact of aflatoxin B1 (AFB1) and DON on cereal famers and the associated issues for farmed animal producers. Ultimately cereal- or animal-based foods contaminated with these mycotoxins, or their masked derivates, can seriously threaten human health.

Both *Aspergillus* and *Fusarium* species have broad host ranges that include important cereal crops. Specifically, maize followed by wheat, are the most frequently reported cereals with the highest concentration of aflatoxins and DON [25]. These mycotoxins can be metabolised, or partially detoxified, within their different plant or animal hosts, creating ‘masked mycotoxins’, which are more difficult to detect. The presence of these mycotoxins and their masked derivatives, within our cereals, have diverse economic impacts and health implications across the food and feed supply chains (Figure 1). Initially, the associated mycotoxin contaminations cause direct economic losses through the reduction of crop yields [2,4]. Harvested cereals that are not lost may then be lost indirectly, as fungal growth and mycotoxins reduce cereal quality and safety, resulting in them being downgraded to animal feed or outright rejected, where cereal farmers can face additional costs associated with waste disposal by landfilling or incineration [18]. Aflatoxin and DON contaminations can occur when cereals are either in the field or during storage. In the field, mycotoxin contamination is influenced by various factors, including the weather, crop variety, and insect damage. For example, elevated atmospheric CO_2_ and crop growth at 20°C/18°C during the day/night cycle results in increased DON contamination [26]. However, aflatoxin is more commonly associated with postharvest contamination during grain storage, while DON is frequently reported as a preharvest in-field contamination issue.

### Aflatoxins

**Figure 2:**
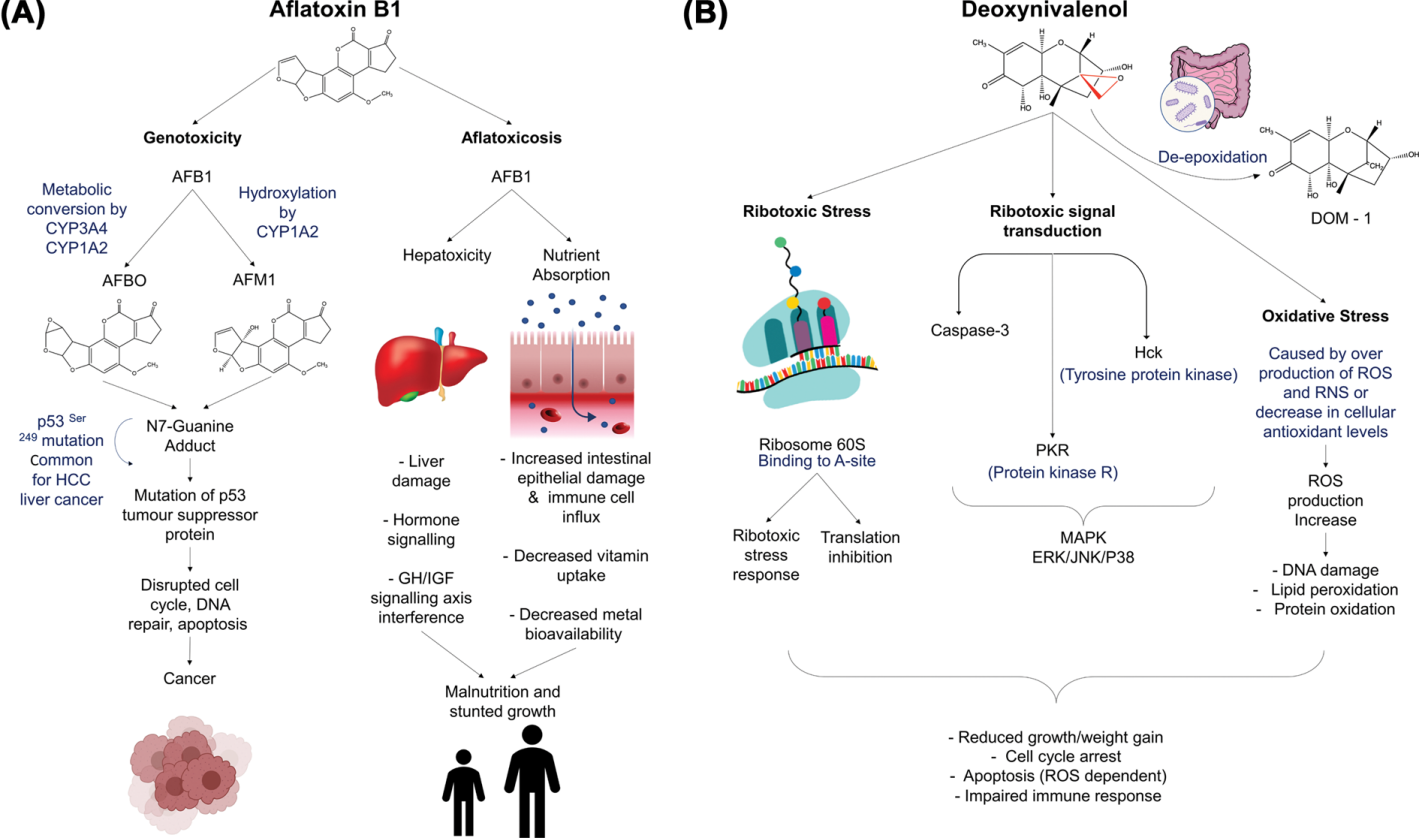
Mechanism of AFB1 induced cancer, malnutrition and stunted growth, and DON induced impairment of immune response, cell cycle arrest, and apoptosis (**A**) AFB1 causes genotoxicity through the conversion and hydroxylation of AFB1 to AFBO and AFM1, resulting in mutation in the tumour-suppression protein p53. AFB1 additionally causes hepatoxicity and interferes with nutrient absorption, resulting in cancer and growth reduction. (**B**) DON can be de-epoxidated into DOM-1 by gut bacteria that is less stable than DON and does not cause the ribotoxic stress response. DON can cause ribotoxic stress and interfere with translation inhibition. DON activates PKR and HcK resulting in increased MAPK. Oxidative stress is caused by increased ROS and RNS, and results in DNA damage, and protein oxidation whereby this leads to cell cycle arrest and apoptosis and impairment of immune response.

Aflatoxin contaminations are predominately caused by *Aspergillus flavus*, which is ubiquitous in nature and agricultural areas [27]. Aflatoxin B1 (AFB1) is regarded as one of the most toxic agents to humans and farmed animals, causing weight loss, immunosuppression, growth impairment, and hepatocellular carcinoma formation [28] (Figure 2A). Specifically, malnutrition can occur when AFB1 consumption results in hepatoxicity through liver damage, interference with GH/IGF signalling and a reduction in hormone signalling, in addition to impacting nutrient absorption in the digestive system. However, the exact mechanism still needs to be fully understood in humans [29–31]. Within the livers of animals and humans, which have eaten AFB1-contaminated food or feed, microsomal mixed-function oxidase enzymes (CYP1A2 and CYP3A4) convert AFB1 into the hydroxylated-derivative AFM1 and other metabolites, including AFB1-8,9-epoxide (AFBO). AFBO and AFM1 confer carcinogenic properties to AFB1, where they conjugate on the N7 position of deoxyguanosine in DNA, forming an AFB1-DNA adduct, 8,9-dihydro-8-(N7-guanyl)-9-hydroxy AFB1 [32,33]. The adduct is unstable, promoting one of two things to occur on the guanine residue: (1) a stable open ring structure forms to make an AFB1-formamidopyridine adduct (AFB1-FAPy), or (2) depurination occurs, causing an increase of free AFB1-N7-guanine [34]. Exposure to AFB1, AFB1-FAPy lesions and the AP site are all known to be associated with 249^Arg^ → 249^Ser^ mutations of the tumour-suppression protein p53 [35–37]. Therefore, AFB1 consumption can result in increased DNA mutations, cell death, and liver cancer development [38,39].

Unsurprisingly, aflatoxin contamination of animal feed increases production costs, due to higher mortality rates, reproductive failures, reduced feed efficiency, and overall quality loss. Aflatoxins also impact the poultry industry, by delaying bird maturity, slowing body weight gain, decreasing egg production, and reducing quality, as eggs may contain AFB1 residues [40]. But egg quality is predominantly impacted via aflatoxins reducing the metabolic potential of the bird’s liver, as proteins and lipids synthesized in the liver are incorporated into the egg yolks [41].

Aflatoxin-contaminated milk products are a particular concern, as cytotoxic AFM1 can be excreted in animal or human breast milk, where it is not denatured by heat or pasteurisation treatments [42]. Consequently, the consumption of AFM1-contaminated milk causes serious child health concerns, including stunted infant growth and increased susceptibility to infectious diseases [43]. AFM1 outbreaks dramatically affect the whole milk supply chain. One example comes from Serbia in 2013, where small farms were most affected, experiencing financial loss due to the lower milk prices they received from dairy companies, plus the cost of additional mycotoxin absorbers and milk testing. Collectively, these impacts lead to decreased demand and the reduced exportation of Serbian milk products [44].

### DON

Fusarium Head Blight and the associated mycotoxins, including DON, are prevalent throughout all cereal growing regions of the world. DON is a trichothecene mycotoxin and exerts its toxicity by binding to ribosomes – particularly the 60S subunit – impairing protein synthesis through the activation of cellular kinases associated with proliferation, differentiation, and apoptosis [45] (Figure 2B). DON can be modified through de-epoxidation by gut microbiota into DOM-1 [46], However, DOM-1 which lacks the epoxy group, is not as stable when binding to ribosomes, and is therefore less toxic [47]. Upon consumption, DON targets innate immune system mononuclear phagocytes [48,49], causes the partial inhibition of protein translation, and activates ribotoxic stress. Translation inhibition can be caused by DON in several ways, such as interfering with the ribosomal peptidyl transferase function resulting in an impairment of initiation and elongation [50,51], through interfering with apoptotic associated pathways causing the degradation of 18S and 28S rRNA [52], and by inhibiting translation through the activation of phosphorylates eIF2a [53]. DON induces ribotoxic stress responses and triggers protein kinase R (PKR) and tyrosine-protein kinase, prior to mitogen-activated protein kinases signalling. However, DOM-1 does not activate the stress response due to the lack of a C12 hydrogen bond on the epoxy group and a hydrogen on uracil U2873 [47,54]. DON exposure also causes the accumulation of reactive oxygen species (ROS), through their overproduction and the decreased production of antioxidants. Increased ROS levels lead to cellular, nuclear and DNA damage, and protein oxidation [55]. Collective these outcomes to DON consumption impact on cell cycle arrest, apoptosis, and has effects on the immune system.

In wheat, DON causes the inhibition of immune responses enabling infection [56]. The consumption of cereal-based food and feed remains the primary route for DON exposure in humans and farmed animals [57]. In mammals, the consumption of DON-contaminated grain causes leukocyte apoptosis and immunosuppression, which in the case of farmed animals causes emesis, anorexia, poor growth, and development [51]. The pig industry is particularly susceptible, as pigs are unable to detoxify DON, resulting in poor growth that delays the time taken to reach their marketable weight, which has serious economic consequences for producers [58]. Furthermore, the quality of edible animal tissues could pose further risks, as some studies have indicated that DON can be passed into animal products that could threaten human health if consumed [57].

Cereals crops can partially detoxify DON through its conjugation with a glucose moiety. This results in the generation of DON-3-β-d-glucoside (DON-3G). Specifically, this glycosylation changes the physicochemical properties of DON, enabling DON-3G to escape the extraction and detection methods commonly used to monitor DON contamination [59]. Although DON-3G is less toxic compared with DON, intestinal bacteria can hydrolyse DON-3G, releasing its toxic DON precursor during mammalian digestion. However, high concentrations of preintestinal bacteria present in ruminants and poultry can also convert DON into the nontoxic de-epoxide derivative DOM-1 [60]. Hence, dietary exposure to DON may be greater than estimated, where its masked forms may also present a serious threat to the safety of cereal-based food and feed, by contributing to elevating total dietary mycotoxin exposure [58].

Therefore, both cereal and farmed-animal producers, plus their associated food and feed industries, bear the costs associated with mycotoxins, which result in consumers experiencing higher food prices [61]. Importantly, the serious health threats from acute and chronic exposure to *Aspergillus* and *Fusarium* mycotoxins may not be disregarded. Understanding how these complex threats may change in our future agricultural environments and food supply chains is a priority.

## Persistent mycotoxin threats to European farming and food safety

Analysis of the EFSA and BIOMIN records for mycotoxins detected over the last decade (2010–2020) shows the persistent risk of aflatoxins and DON in our main cereal crops (barley, maize, oat, rice, and wheat) in both our food and animal feed sectors, raising concerns associated with chronic exposure, masked toxins, and cocontaminations.

### Aflatoxin and DON contamination of food cereals

**Figure 3:**
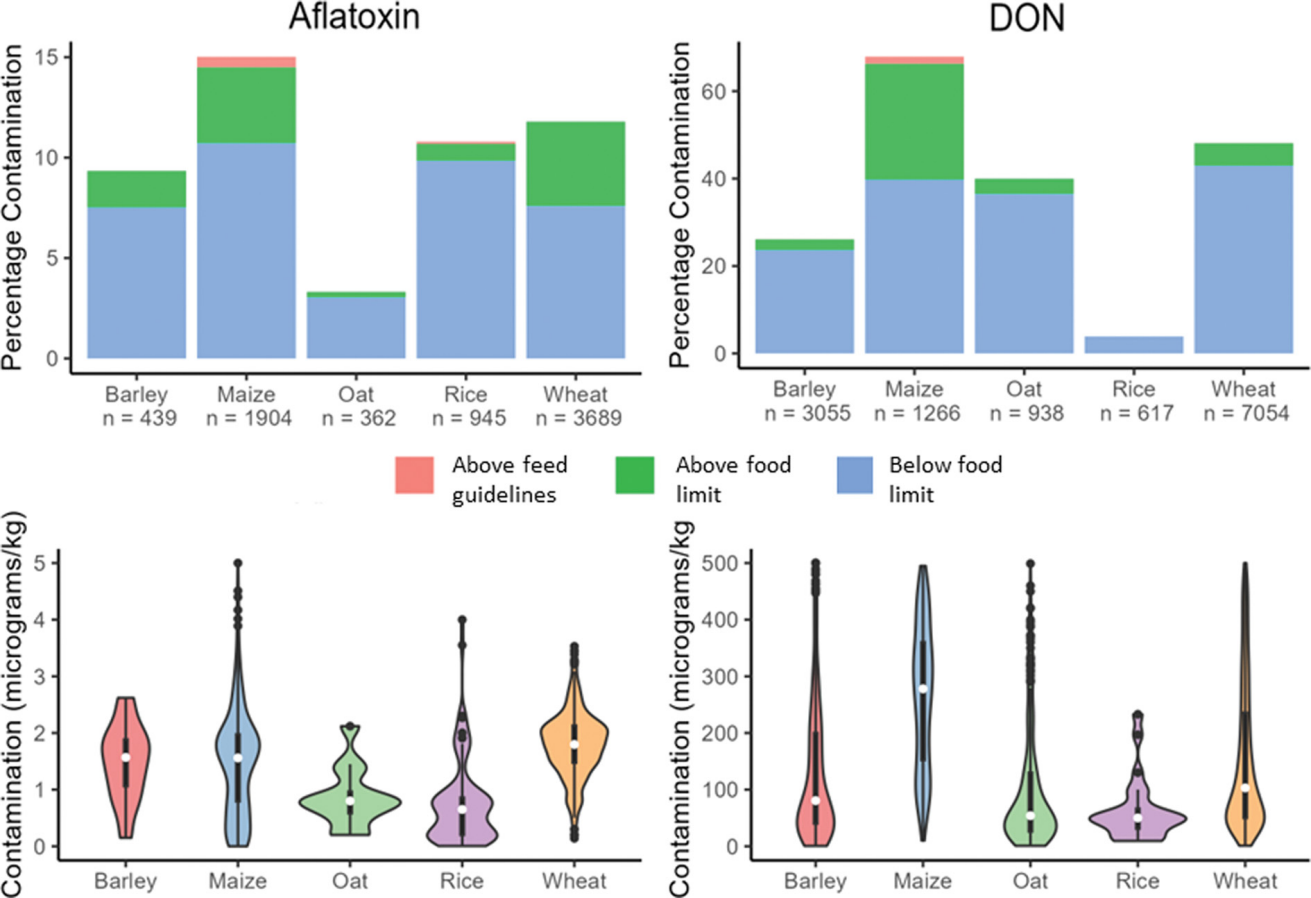
Contamination levels of food grains differ by grain for DON and aflatoxin Contamination data are derived from EFSA records spanning from 2010 to 2020. The percent of contaminated samples below the regulatory limit for food (blue), above the food limit (green), and above the higher feed guideline/recommendation (red), with the total number of samples tested (*n*) for each cereal. Violin plots outlining the distribution of mycotoxin concentrations (µg mycotoxin per kg of grain) with superimposed boxplots displaying medians, interquartile ranges, and outliers. Due to a small number of extreme outliers for maize (as high as 790 µg/kg for aflatoxin and 32300 µg/kg for DON), y-axes were limited to the ranges shown.

DON was frequently detected in all food cereals, with 67.93% of maize and 48.16% of wheat contaminated (Figure 3). Of particular concern were the high average levels of DON found within maize (1448.1 µg/kg) and wheat (359.96 µg/kg), where 28.20% and 5.22% of samples had DON levels above the safety threshold (750 µg/kg) for human food, respectively, and 1.66% of maize was also above the guide for animal feed (8000 µg/kg) [19,20]. The exception was rice where only 3.89% contained DON and none was above the safe limit for human consumption.

Aflatoxin was also detected in all food cereals, but at a lower frequency than DON contamination, with 15.02% of maize and 11.79% of wheat being contaminated (Figure 3). At less than 5 µg/kg, the levels of aflatoxin contamination were also comparatively lower than DON contaminations. However, the safe food (2 µg/kg) and animal feed (20 µg/kg) thresholds for aflatoxin are much lower; therefore, aflatoxin contamination still results in 4.31% maize and 4.20% wheat being above the limit for food and 0.53% of maize also being above the limit for animal feed [19]. Contrasting DON contaminations where rice was not at risk, 10.79% of rice contained aflatoxins, with 0.95% of contaminations being above the safe food limit. Of all the cereals assessed, oats were least at risk of aflatoxin.

Therefore, our food cereals frequently contain tolerated levels of aflatoxin and DON, but this poses a health concern related to chronic mycotoxin exposure. Importantly, a significant proportion of food is consistently downgraded to animal feed, due to the detection of high contamination levels. For DON contamination across Europe in 2010–2019, Johns et al. (2022) estimated that 75 million tonnes of wheat (5% of food wheat) exceeded the 750 µg/kg limit, where its downgrading to animal feed equated to a loss €3 billion [62]. Here, we showed that aflatoxins caused the downgrading of 4.2% food wheat between 2010 and 2020, potentially equating to an additional €2.5 billion lost. This clearly demonstrates the substantial economic impact of just two mycotoxins on cereal farming, an impact that was extenuated between 2021–2023, when food and feed wheat prices have risen from ∼€200 to ∼€300 per tonne. However, it also suggests the potential for our cereals to be cocontaminated with aflatoxins and DON at the same time, which could have largely uncharacterised human and animal health implications. Similarly, Johns et al. (2022) revealed that wheat is increasing cocontaminated with multiple different *Fusarium* mycotoxins, predominantly DON, zearalenone, and T-2 [62]. Therefore, greater understanding of the health implications of cocontaminations with multiple mycotoxins, and how these risks may change in future climatic scenarios is essential.

### Aflatoxin and DON contamination of cereals for animal feed

**Figure 4:**
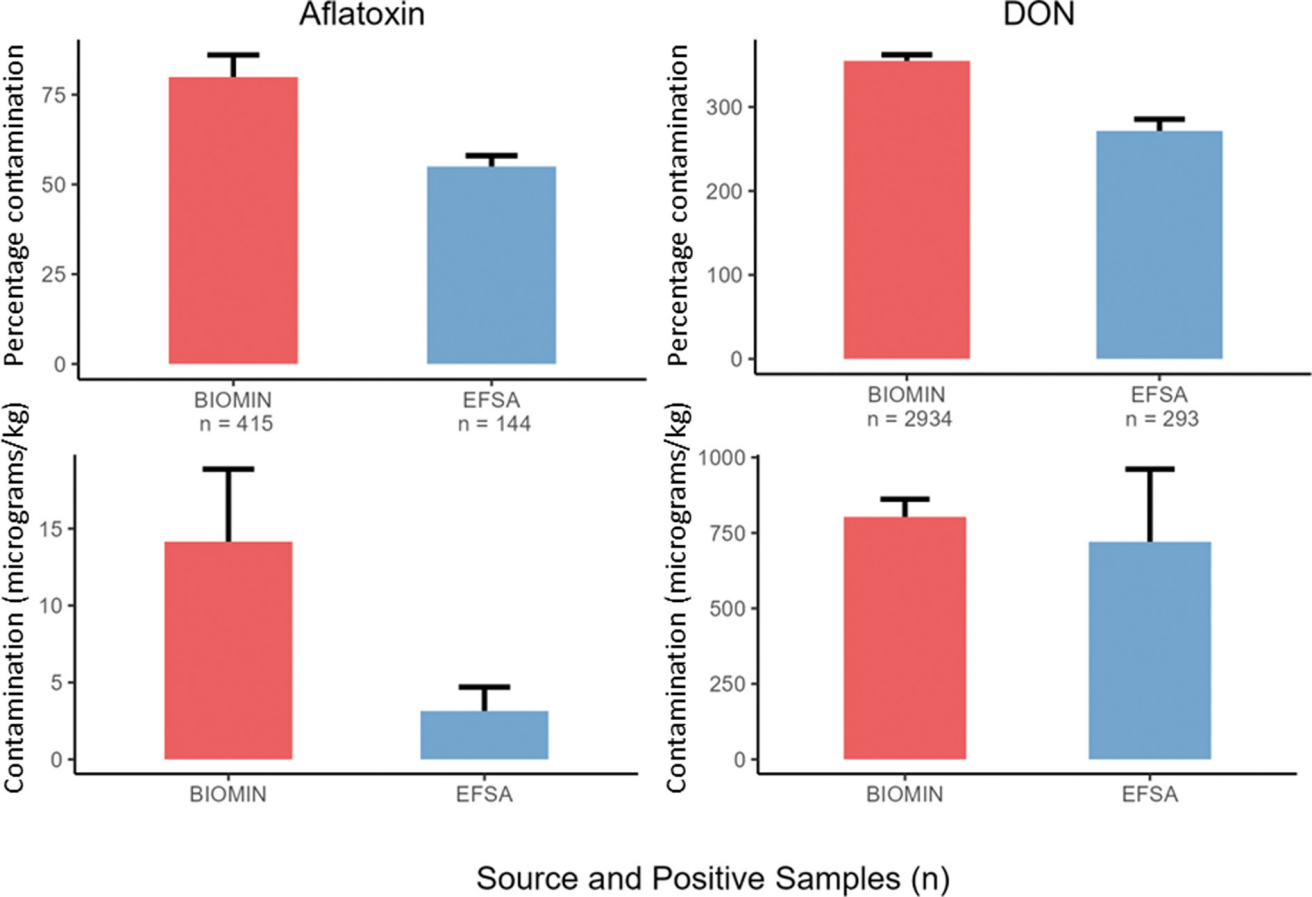
Mycotoxin contamination of animal feed maize exceeds contamination for food maize Bar plots depicting the percentage and number (*n*) of samples containing aflatoxin and DON and the mean concentration (µg mycotoxin per kg of grain) of each mycotoxin. Contamination data for European feed maize from 2016 to 2022 were taken from BIOMIN GmbH annual mycotoxin reports and EFSA mycotoxin surveillance data were used for food cereals. Two outliers with excessively high contamination (790 µg/kg aflatoxin, 23500 µg/kg DON) were omitted from analysis. Error bars represent standard deviation around the mean.

High levels of aflatoxin and DON contamination in food cereals demonstrate that maize is particularly at risk (Figure 3). Comparisons of mycotoxin contaminations in EFSA and BIOMIN data show the different threats posed to our food and feed supply chains (Figure 4). Aflatoxin and DON contamination frequencies were only slightly increased in maize destined for animal feed. DON contamination levels were comparable between food and feed, around ∼750 µg/kg limit for human consumption [17]. However, at ∼15 µg/kg, aflatoxin levels were significantly higher in maize destined for animal feed, which was significantly higher than the limit permitted for human consumption (2 µg/kg), dairy or young animals (5 µg/kg), and approaching the 20 µg/kg guide for other animals [4,58,63]. This again indicates the elevated risk of aflatoxin and DON mycotoxins in maize, while also demonstrating their diverse threats to our feed supply chains, where mycotoxins can undergo bioconversion into mixtures of masked toxins that are not easily detected using traditional testing systems.

## How our changing agricultural environments influence the mycotoxin threat

Our agricultural environments are changing, and this is reflected in changing fungal crop disease pressures and mycotoxin contaminations. Driven by climate change and altered agronomic practices fungal pathogens are on the move, due to the geographic expansion of favourable conditions and hosts [64]. This can contribute to changing patterns in crop contamination, through the creation of increasingly favourable conditions for existing contaminations, or through the arrival of new mycotoxigenic fungal contaminants [62]. Next, we discuss how future environments may influence *Aspergillus* and *Fusarium* mycotoxin contaminations of cereals.

**Figure 5:**
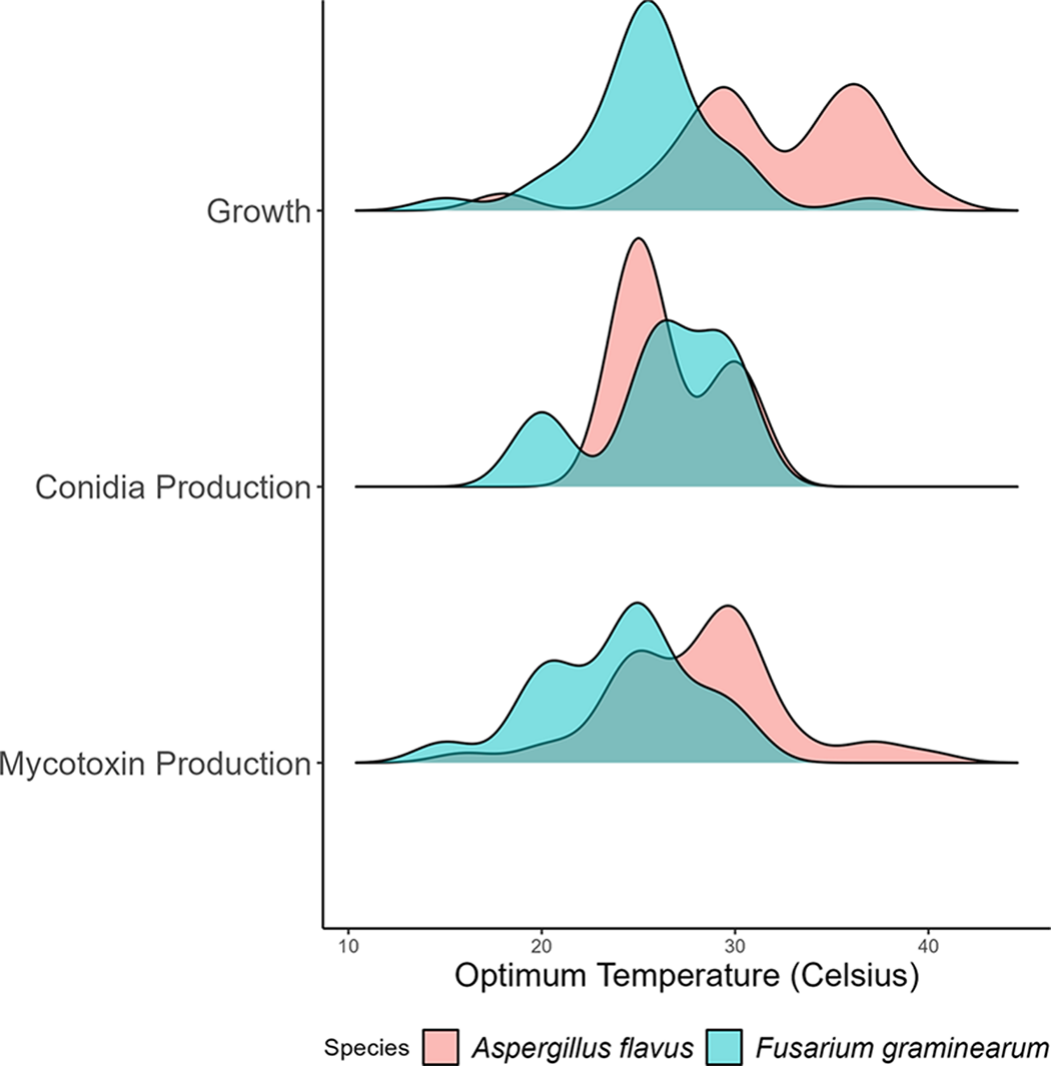
The optimum temperatures for key life processes for *A. flavus* and *F. graminearum* Key life processes shown are the production of respective mycotoxins (aflatoxin and DON), mycelial growth in culture, and asexual conidia production. The density curves represent the distribution of published temperature optima. The height of a curve corresponds to the proportion of reports of optima at a given temperature.

**Figure 6:**
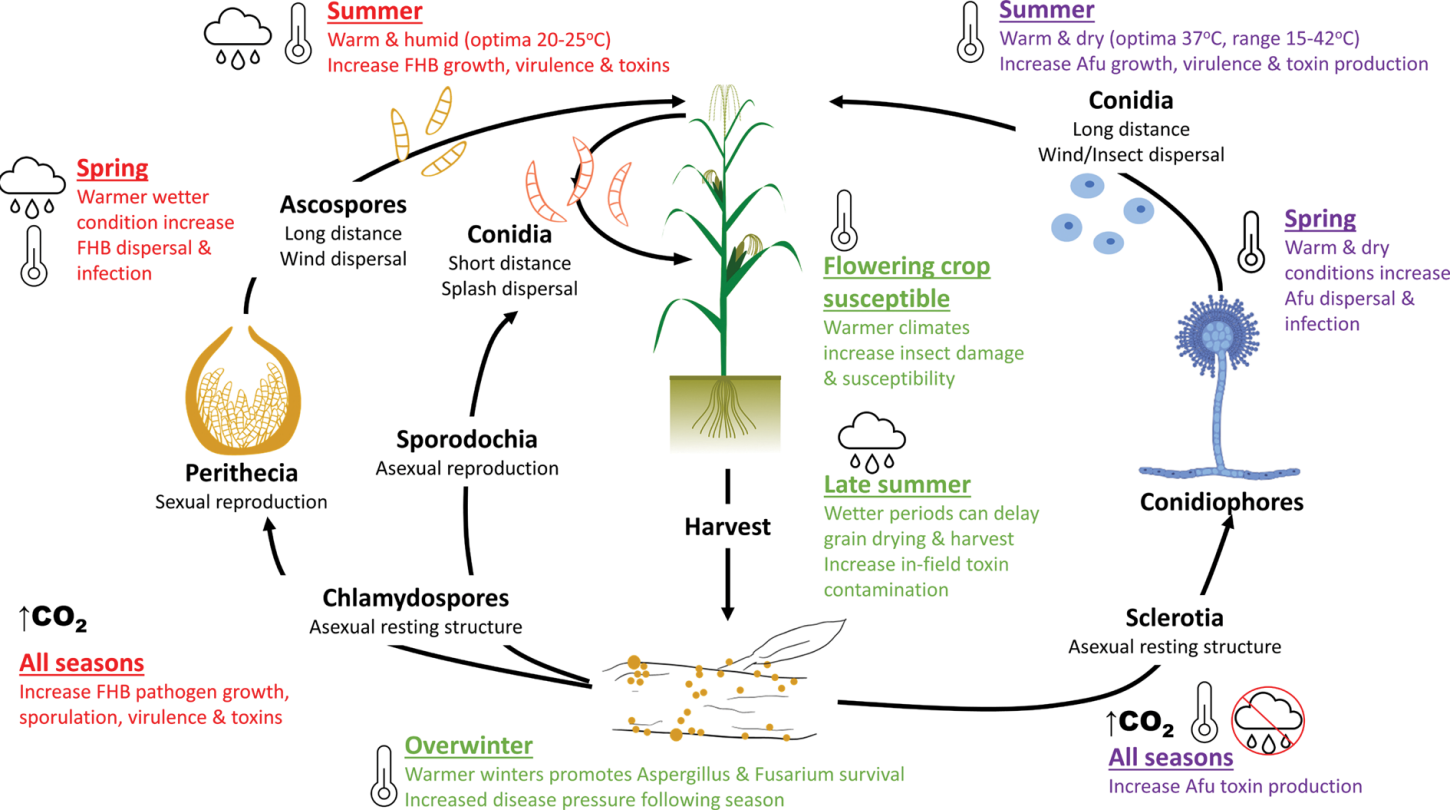
Climate change impacts on risk of cereal contamination by *A. flavus* and *F. graminearum* Influence of climatic conditions on life process is shown in red (*F. graminearum*), purple (*A. flavus*), and green (both). Climate change is predicted to drive conditions in northern Europe towards warmer wetter environments favoured by *F. graminearum*. In contrast, southern Europe is expected to become hotter and dryer, as favoured by *A. flavus*. In both systems, elevated CO_2_ is associated with increased mycotoxin contamination. Climate change projections may therefore allow for increased contamination of our cereals by mycotoxigenic fungi.

Optimal conditions for key fungal processes, namely mycelial growth, dissemination by sporulation, and mycotoxin biosynthesis, vary depending on the strain and nutritional environment assessed. However, the distribution of the outcome from published studies can reveal the optimal environments for *A. flavus* and *F. graminearum* (Figure 5) and help us to understand how changing environments could lead to the increased risk of cereal infection and mycotoxin contamination (Figure 6).

### Optimum T, *a*_w_, CO_2_ conditions for growth, dissemination, and mycotoxin production

*A. flavus* grows at temperatures from 10°C to 48°C [65]. However, the distribution of optima suggests between 28°C and 37°C best supports fungal growth (Figure 5). Unlike many other fungi, *A. flavus* is xerophilic and thus well adapted to grow at low water activity (*a*_w_) with some strains growing at conditions as low as 0.73 *a*_w_ [66]. The abundant production and dissemination of airborne conidia and their subsequent germination is optimal at 25°C. Aflatoxin production also occurs at a wide range of temperatures, with an optimum between 28°C and 30°C [67]. Further to this, aflatoxins are predicted to become a major food safety issue in maize, especially in countries where a rise in temperature, together with increased CO_2_ levels, and droughts are predicted to occur [68]. These traits allow *A. flavus* to thrive and outcompete other fungi in hot dry conditions, which can promote in-field cereal contaminations (Figure 6) or spoilage during storage.

The optimum growth temperature for *F. graminearum* is between 20°C and 25°C, with scant growth at low (5°C) and high (35°C) temperatures [69] (Figure 5), reflecting its geographic distribution across temperate-cereal-growing regions. The production of splash-dispersed asexual conidia favours slightly higher temperatures [10,70] with an optimum estimated at 28°C [71,72]. Similarly, perithecial maturation (20–25°C) and the discharge of wind-blown sexual ascospores (25–28°C) favour warm temperatures. This reflects the fine orchestration of sexual and asexual reproduction cycles with seasonal temperature changes [70] (Figure 6). DON production occurs over a narrow range (15–30°C), with an optimum of 25–28°C [73,74]. As a hydrophilic fungus, *F. graminearum* thrives in wetter environments [75,76]. Mycelial growth only occurs above 0.9 *a*_w_ [66] with optima between 0.980 and 0.995 *a*_w_ [77]. Splashed-dispersed conidia intuitively require higher *a*_w_ than wind-dispersed ascospores and favour intense rainfall coinciding with crop susceptibility at anthesis [10,78]. Yet while ascospore dispersal can increase after a brief dry period, moisture also reliably predicts airborne ascospore prevalence, indicating that high moisture is indispensable for both ascospore and conidia dissemination [10]. DON production, as with temperature, occurs over a narrower *a*_w_ range (0.93–0.995 *a*_w_) peaking at 0.995 *a*_w_ [66,73]. After fungal acclimation to the IPCC scenario predicted for 2100, elevated CO_2_ was also found to promote cereal disease severity, impacting on seed number and weight [79]. Significant increases in DON contamination have also been observed in maize and wheat plants grown under increased CO_2_ levels [26]. This may owe to greater host susceptibility, as elevated CO_2_ has been shown to downregulate the defensive phenylpropanoid pathway in wheat [10]. These traits reflect why *F. graminearum* is a threat in warm moist environments, where it promotes in-field cereal contaminations (Figure 6).

### How optimal conditions relate to Europe geographic mycotoxin threats

The two species presented illustrate how fungal pathogens occupy unique ecological niches. The optimum growth and mycotoxin production conditions depict clear differences, with *F. graminearum* favoured by warm wet and *A. flavus* by hot dry environments (Figure 6). Therefore, climatic changes may differentially influence the threat of *Aspergillus* and *Fusarium* mycotoxins across Europe.

In the last decade, *Fusarium* mycotoxins in cereals have increased, particularly in southern Europe [62], which may reflect the alignment of specific geographic environments with optimal conditions for contaminations to occur. However, European temperatures are expected to continue to increase by 3–5°C under climate change projections, with differing effects by region [80]. Southern Europe is predicted to experience increased droughts, while Northern Europe may see greater precipitation; with all trends projected to grow more severe [81]. The future warmer springs and increased precipitation in Northern Europe are likely to favour *Fusarium* outbreaks and mycotoxin contaminations [80], while climate change-induced warmer nights (+3°C) are conditions shown to increase *Fusarium* disease severity and DON production [82,83]. In contrast, as hot and dry episodes become more prevalent in southern Europe, this will favour increased *Aspergillus* infections and aflatoxin contamination. Climatic events, such as in northern Italy, have already been seen throughout the last decade in maize-growing regions prior to aflatoxin outbreaks [80]. Furthermore, outbreaks are predicted to become more of a threat with climate change, especially in the Mediterranean basin, which was highlighted to be warming 20% faster than the global average [84].

Climate change, therefore, threatens to exacerbate the intensity and extent of mycotoxin contamination in Europe, demanding concerted efforts in the surveillance and control of mycotoxigenic fungal pathogens, to mitigate the risks they pose to sustainable safe cereal production.

## Summary

- Fungal pathogens, including toxigenic *Aspergillus* and *Fusarium* species, consistently contaminate our food and feed cereals, producing harmful mycotoxins, threatening human and animal health.
- Despite established monitoring systems and disease control approaches, mycotoxin contaminations consistently threaten to our food security, through the understudied impacts of: (i) chronic mycotoxin exposure, (ii) the underestimated dietary intake of masked mycotoxins, and (iii) cocontaminations with multiple mycotoxins.
- Mycotoxins have profound economic consequences for cereal and farmed-animal producers, plus their associated food and feed industries, which results higher food prices for consumers.
- Changing environments threaten to exacerbate the extent and intensity of *Aspergillus* and *Fusarium* mycotoxin contaminations of cereals, highlighting the need for concerted efforts to understand and mitigate increased risks.

## Open Access

Open access for the present article was enabled by the participation of University of Bath in an all-inclusive *Read & Publish* agreement with Portland Press and the Biochemical Society under a transformative agreement with JISC.

## Abbreviations

AFB1: aflatoxin B1
AFB1-FAPy: AFB1-formamidopyridine adduct
AFBO: AFB1-8,9-epoxide
DON: deoxynivalenol
DON-3G: DON-3-β-d-glucoside
EFSA: European Food Safety Agency
PKR: protein kinase R
ROS: reactive oxygen species

## References

[1] Bennett J.W. and Klich M. (2003) Mycotoxins. Clin. Microbiol. Rev. 16, 497 10.1128/CMR.16.3.497-516.2003 12857779 PMC164220

[2] Navale V., Vamkudoth K.R., Ajmera S. and Dhuri V. (2021) *Aspergillus* derived mycotoxins in food and the environment: prevalence, detection, and toxicity. Toxicol. Rep. 8, 1008–1030 10.1016/j.toxrep.2021.04.013 34408970 PMC8363598

[3] Pitt J.I. and Miller J.D. (2017) A concise history of mycotoxin research. J. Agric. Food Chem. 65, 7021–7033 10.1021/acs.jafc.6b04494 27960261

[4] Eskola M., Altieri A. and Galobart J. (2018) Overview of the activities of the European Food Safety Authority on mycotoxins in food and feed. World Mycotoxin J. 11, 277–289 10.3920/WMJ2017.2270

[5] Jallow A., Xie H.L., Tang X.Q., Qi Z. and Li P.W. (2021) Worldwide aflatoxin contamination of agricultural products and foods: from occurrence to control. Compr. Rev. Food Sci. Food Saf. 20, 2332–2381 10.1111/1541-4337.12734 33977678

[6] Ojiambo P.S., Battilani P., Cary J.W., Blum B.H. and Carbone I. (2018) Cultural and genetic approaches to manage aflatoxin contamination: recent insights provide opportunities for improved control. Phytopathology 108, 1024–1037 10.1094/PHYTO-04-18-0134-RVW 29869954

[7] Sarma U.P., Bhetaria P.J., Devi P. and Varma A. (2017) Aflatoxins: implications on health. Indian J. Clin. Biochem. 32, 124–133 10.1007/s12291-017-0649-2 28428686 PMC5382086

[8] Probst C., Bandyopadhyay R. and Cotty P.J. (2014) Diversity of aflatoxin-producing fungi and their impact on food safety in sub-Saharan Africa. Int. J. Food Microbiol. 174, 113–122 10.1016/j.ijfoodmicro.2013.12.010 24480188

[9] Food Standards Agency. (2021) Mycotoxins, Food Standards Agency, London. https://www.food.gov.uk/business-guidance/mycotoxins

[10] Vaughan M., Backhouse D. and Del Ponte E.M. (2016) Climate change impacts on the ecology of *Fusarium graminearum* species complex and susceptibility of wheat to *Fusarium* head blight: a review. World Mycotoxin J. 9, 685–700 10.3920/WMJ2016.2053

[11] Kant P., Reinprecht Y., Martin C., Islam R. and Pauls K. (2019) Disease resistance. In Comprehensive Biotechnology, pp. 789–805, Elsevier, Amsterdam, The Netherlands

[12] Dean R., Van Kan J.A.L., Pretorius Z.A., Hammond-Kosack K.E., Di Pietro A., Spanu P.D. et al. (2012) The top 10 fungal pathogens in molecular plant pathology. Mol. Plant Pathol. 13, 414–430 10.1111/j.1364-3703.2011.00783.x 22471698 PMC6638784

[13] Desjardins A.E. and Proctor R.H. (2007) Molecular biology of *Fusarium* mycotoxins. Int. J. Food Microbiol. 119, 47–50 10.1016/j.ijfoodmicro.2007.07.024 17707105

[14] Eskola M., Kos G., Elliott C.T., Hajslova J., Mayar S. and Krska R. (2020) Worldwide contamination of food-crops with mycotoxins: validity of the widely cited ‘FAO estimate’ of 25%. Crit. Rev. Food Sci. Nutr. 60, 2773–2789 10.1080/10408398.2019.1658570 31478403

[15] Dunwell J.M. (2014) Transgenic cereals: current status and future prospects. J. Cereal Sci. 59, 419–434 10.1016/j.jcs.2013.08.008

[16] Alshannaq A. and Yu J.H. (2017) Occurrence, toxicity, and analysis of major mycotoxins in food. Int. J. Environ. Res. Public Health 14, 20 10.3390/ijerph14060632 PMC5486318 28608841

[17] European Commission (2006) Commission Regulation (EC) 1881/2006. Legislation.gov. https://www.legislation.gov.uk/eur/2006/1881

[18] Alassane-Kpembi I., Schatzmayr G., Taranu I., Marin D., Puel O. and Oswald I.P. (2017) Mycotoxins co-contamination: methodological aspects and biological relevance of combined toxicity studies. Crit. Rev. Food Sci. Nutr. 57, 3489–3507 10.1080/10408398.2016.1140632 26918653

[19] EUR-Lex. (2006) Commission Regulation (EC) No 1881/2006, European Union. EUR-Lex. https://eur-lex.europa.eu/LexUriServ/LexUriServ.do?uri=OJ:L:2006:364:0005:0024:EN:PDF

[20] Agriculture and Horticulture Development Board. *Fusarium* and Microdochium in Cereals Agriculture and Horticulture Development Board, Warwickshire. https://ahdb.org.uk/knowledge-library/Fusarium-and-microdochium-in-cereals#h25

[21] Ferrigo D., Raiola A. and Causin R. (2016) *Fusarium* toxins in cereals: occurrence, legislation, factors promoting the appearance and their management. Molecules 21, 35 10.3390/molecules21050627 PMC6274039 27187340

[22] Kumar A., Pathak H., Bhadauria S. and Sudan J. (2021) Aflatoxin contamination in food crops: causes, detection, and management: a review. Food Production Process. Nutr. 3, 9 10.1186/s43014-021-00064-y

[23] Ederli L., Beccari G., Tini F., Bergamini I., Bellezza I., Romani R. et al. (2021) Enniatin B and deoxynivalenol activity on bread wheat and on *Fusarium* Species development. Toxins 13, 17 10.3390/toxins13100728 PMC8538094 34679021

[24] Gruber-Dorninger C., Jenkins T. and Schatzmayr G. (2019) Global mycotoxin occurrence in feed: a ten-year survey. Toxins 11, 25 10.3390/toxins11070375 31252650 PMC6669473

[25] Palumbo R., Crisci A., Venancio A., Abrahantes J.C., Dorne J.L., Battilani P. et al. (2020) Occurrence and co-occurrence of mycotoxins in cereal-based feed and food. Microorganisms 8, 17 10.3390/microorganisms8010074 PMC7023405 31947721

[26] Hay W.T., McCormick S.P. and Vaughan M.M. (2021) Effects of atmospheric CO2 and temperature on wheat and corn susceptibility to *Fusarium graminearum* and deoxynivalenol contamination. Plants-Basel 10, 13 10.3390/plants10122582 PMC8709488 34961056

[27] Perrone G., Gallo A. and Logrieco A.F. (2014) Biodiversity of *Aspergillus* section *Flavi* in Europe in relation to the management of aflatoxin risk. Front. Microbiol. 5, 5 10.3389/fmicb.2014.00377 25101075 PMC4104701

[28] Dhanasekaran D., Shanmugapriya S., Thajuddin N. and Panneerselvam A. (2011) Aflatoxins and aflatoxicosis in human and animals. In Aflatoxins(Ramón Gerardo G.-G., ed.), pp. 221–254, IntechOpen, Rijeka 10.5772/22717

[29] Rushing B.R. and Selim M.I. (2019) Aflatoxin B1: a review on metabolism, toxicity, occurrence in food, occupational exposure, and detoxification methods. Food Chem. Toxicol. 124, 81–100 10.1016/j.fct.2018.11.047 30468841

[30] Knipstein B., Huang J.S., Barr E., Sossenheimer P., Dietzen D., Egner P.A. et al. (2015) Dietary aflatoxin-induced stunting in a novel rat model: evidence for toxin-induced liver injury and hepatic growth hormone resistance. Pediatr. Res. 78, 120–127 10.1038/pr.2015.84 25938735 PMC4506701

[31] Kihara T., Matsuo T., Sakamoto M., Yasuda Y., Yamamoto Y. and Tanimura T. (2000) Effects of prenatal aflatoxin B1 exposure on behaviors of rat offspring. Toxicol. Sci. 53, 392–399 10.1093/toxsci/53.2.392 10696787

[32] Croy R., Essigmann J., Reinhold V. and Wogan G. (1978) Identification of the principal aflatoxin B1-DNA adduct formed in vivo in rat liver. Proc. Natl. Acad. Sci. U.S.A. 75, 1745–1749 10.1073/pnas.75.4.1745 273905 PMC392416

[33] Martin C.N. and Garner R.C. (1977) Aflatoxin B-oxide generated by chemical or enzymic oxidation of aflatoxin B 1 causes guanine substitution in nucleic acids. Nature 267, 863–865 10.1038/267863a0 895848

[34] Groopman J.D., Croy R.G. and Wogan G.N. (1981) In vitro reactions of aflatoxin B1-adducted DNA. Proc. Natl. Acad. Sci. U.S.A. 78, 5445–5449 10.1073/pnas.78.9.5445 6795633 PMC348762

[35] Aguilar F., Hussain S.P. and Cerutti P. (1993) Aflatoxin B1 induces the transversion of G–>T in codon 249 of the p53 tumor suppressor gene in human hepatocytes. PNAS 90, 8586–8590 10.1073/pnas.90.18.8586 8397412 PMC47402

[36] Bailey E.A., Iyer R.S., Stone M.P., Harris T.M. and Essigmann J.M. (1996) Mutational properties of the primary aflatoxin B1-DNA adduct. Proc. Natl. Acad. Sci. U.S.A. 93, 1535–1539 10.1073/pnas.93.4.1535 8643667 PMC39975

[37] Smela M.E., Hamm M.L., Henderson P.T., Harris C.M., Harris T.M. and Essigmann J.M. (2002) The aflatoxin B1 formamidopyrimidine adduct plays a major role in causing the types of mutations observed in human hepatocellular carcinoma. Proc. Natl. Acad. Sci. U.S.A. 99, 6655–6660 10.1073/pnas.102167699 12011430 PMC124458

[38] Brown K.L., Deng J.Z., Iyer R.S., Iyer L.G., Voehler M.W., Stone M.P. et al. (2006) Unraveling the aflatoxin− FAPY conundrum: structural basis for differential replicative processing of isomeric forms of the formamidopyrimidine-type DNA adduct of aflatoxin B1. J. Am. Chem. Soc. 128, 15188–15199 10.1021/ja063781y 17117870 PMC2693076

[39] Marchese S., Polo A., Ariano A., Velotto S., Costantini S. and Severino L. (2018) Aflatoxin B1 and M1: biological properties and their involvement in cancer development. Toxins 10, 19 10.3390/toxins10060214 PMC6024316 29794965

[40] Diaz-Zaragoza M., Carvajal-Moreno M., Mendez-Ramirez I., Chilpa-Galvan N.C., Avila-Gonzalez E. and Flores-Ortiz C.M. (2014) Aflatoxins, hydroxylated metabolites, and aflatoxicol from breast muscle of laying hens. Poult. Sci. 93, 3152–3162 10.3382/ps.2014-04240 25352677

[41] Monson M.S., Coulombe R.A. and Reed K.M. (2015) Aflatoxicosis: lessons from toxicity and responses to aflatoxin B1 in poultry. Agriculture 5, 742–777 10.3390/agriculture5030742

[42] Peles F., Sipos P., Gyori Z., Pfliegler W.P., Giacometti F., Serraino A. et al. (2019) Adverse effects, transformation and channeling of aflatoxins into food raw materials in livestock. Front. Microbiol. 10, 26 10.3389/fmicb.2019.02861 31921041 PMC6917664

[43] Winter G. and Pereg L. (2019) A review on the relation between soil and mycotoxins: effect of aflatoxin on field, food and finance. Eur. J. Soil Sci. 70, 882–897 10.1111/ejss.12813

[44] Popovic R., Radovanov B. and Dunn J.W. (2017) Food scare crisis: the effect on Serbian dairy market. Int. Food Agribusiness Management Rev. 20, 113–127 10.22434/IFAMR2015.0051

[45] Diamond M., Reape T.J., Rocha O., Doyle S.M., Kacprzyk J., Doohan F.M. et al. (2013) The *Fusarium* Mycotoxin deoxynivalenol can inhibit plant apoptosis-like programmed cell death. PloS ONE 8, 8 10.1371/journal.pone.0069542 PMC3724914 23922734

[46] Yoshizawa T., Takeda H. and Ohi T. (1983) Structure of a novel metabolite from deoxynivalenol, a trichothecene mycotoxin, in animals. Agric. Biol. Chem. 47, 2133–2135 10.1271/bbb1961.47.2133

[47] Pierron A., Mimoun S., Murate L.S., Loiseau N., Lippi Y., Bracarense A. et al. (2016) Microbial biotransformation of DON: molecular basis for reduced toxicity. Sci. Rep. 6, 13 10.1038/srep29105 27381510 PMC4933977

[48] Pestka J.J. and Smolinski A.T. (2005) Deoxynivalenol: toxicology and potential effects on humans. J. Toxicol. Environ. Health-Part B-Critical Rev. 8, 39–69 10.1080/10937400590889458 15762554

[49] Pierron A., Mimoun S., Murate L.S., Loiseau N., Lippi Y., Bracarense A. et al. (2016) Intestinal toxicity of the masked mycotoxin deoxynivalenol-3-beta-d-glucoside. Arch. Toxicol. 90, 2037–2046 10.1007/s00204-015-1592-8 26404761

[50] Ehrlich K.C. and Daigle K.W. (1987) Protein synthesis inhibition by 8-oxo-12,13-epoxytrichothecenes. Biochim. Biophys. Acta 923, 206–213 10.1016/0304-4165(87)90005-5 3814614

[51] Pestka J.J. (2010) Deoxynivalenol: mechanisms of action, human exposure, and toxicological relevance. Arch. Toxicol. 84, 663–679 10.1007/s00204-010-0579-8 20798930

[52] He K.Y., Zhou H.R. and Pestka J.J. (2012) Targets and intracellular signaling mechanisms for deoxynivalenol-induced ribosomal RNA cleavage. Toxicol. Sci. 127, 382–390 10.1093/toxsci/kfs134 22491426 PMC3355321

[53] Zhou H.R., Lau A.S. and Pestka J.J. (2003) Role of double-stranded RNA-activated protein kinase R (PKR) in deoxynivalenol-induced ribotoxic stress response. Toxicol. Sci. 74, 335–344 10.1093/toxsci/kfg148 12773753

[54] Pestka J.J. (2007) Deoxynivalenol: toxicity, mechanisms and animal health risks. Anim. Feed Sci. Technol. 137, 283–298 10.1016/j.anifeedsci.2007.06.006

[55] Mishra S., Dwivedi P.D., Pandey H.P. and Das M. (2014) Role of oxidative stress in deoxynivalenol induced toxicity. Food Chem. Toxicol. 72, 20–29 10.1016/j.fct.2014.06.027 25010452

[56] Brown N.A., Evans J., Mead A. and Hammond-Kosack K.E. (2017) A spatial temporal analysis of the *Fusarium graminearum* transcriptome during symptomless and symptomatic wheat infection. Mol. Plant Pathol. 18, 1295–1312 10.1111/mpp.12564 28466509 PMC5697668

[57] Goyarts T., Danicke S., Valenta H. and Ueberschar K.H. (2007) Carry-over of *Fusarium* toxins (deoxynivalenol and zearalenone) from naturally contaminated wheat to pigs. Food Addit. Contam. 24, 369–380 10.1080/02652030600988038 17454110

[58] Sayyari A., Faeste C.K., Hansen U., Uhlig S., Framstad T., Schatzmayr D. et al. (2018) Effects and biotransformation of the mycotoxin deoxynivalenol in growing pigs fed with naturally contaminated pelleted grains with and without the addition of Coriobacteriaceum DSM 11798. Food Addit. Contam. 35, 1394–1409 10.1080/19440049.2018.1461254 29701502

[59] Alizadeh A., Braber S., Akbari P., Kraneveld A., Garssen J. and Fink-Gremmels J. (2016) Deoxynivalenol and its modified forms: are there major differences? Toxins 8, 16 10.3390/toxins8110334 27854268 PMC5127130

[60] Maresca M. (2013) From the gut to the brain: journey and pathophysiological effects of the food-associated trichothecene mycotoxin deoxynivalenol. Toxins 5, 784–820 10.3390/toxins5040784 23612752 PMC3705292

[61] Wu F. (2006) Mycotoxin reduction in Bt corn: potential economic, health, and regulatory impacts. Transgenic Res. 15, 277–289 10.1007/s11248-005-5237-1 16779644

[62] Johns L.E., Bebber D.P., Gurr S.J. and Brown N.A. (2022) Emerging health threat and cost of *Fusarium* mycotoxins in European wheat. Nat. Food 3, 1014 10.1038/s43016-022-00655-z 37118304

[63] European Feed and Food Ingredient Safety Certification. (2015) European feed ingredients safety code, EFISC. https://www.efisc-gtp.eu/data/1433337461EFISC-%20Code%20of%20good%20practice%20aflatoxin%20monitoring%20version%201.1%20clean.pdf

[64] Bebber D.P., Ramotowski M.A.T. and Gurr S.J. (2013) Crop pests and pathogens move polewards in a warming world. Nat. Climate Change 3, 985–988 10.1038/nclimate1990

[65] Pitt J. and Hocking A.D. (2006) *Aspergillus* and related teleomorphs. In Food spoilage microorganisms. Fungi and Food Spoilage, pp. 451–487, Springer, Cambridge, UK

[66] Hope R., Aldred D. and Magan N. (2005) Comparison of environmental profiles for growth and deoxynivalenol production by *Fusarium culmorum* and F-*graminearum* on wheat grain. Lett. Appl. Microbiol. 40, 295–300 10.1111/j.1472-765X.2005.01674.x 15752221

[67] Obrian G.R., Georgianna D.R., Wilkinson J.R., Yu J., Abbas H.K., Bhatnagar D. et al. (2007) The effect of elevated temperature on gene transcription and aflatoxin biosynthesis. Mycologia 99, 232–239 10.1080/15572536.2007.11832583 17682776

[68] Moretti A., Pascale M. and Logrieco A.F. (2019) Mycotoxin risks under a climate change scenario in Europe. Trends Food Sci. Technol. 84, 38–40 10.1016/j.tifs.2018.03.008

[69] Moreno-Amores J., Michel S., Loschenberger F. and Buerstmayr H. (2020) Dissecting the contribution of environmental influences, plant phenology, and disease resistance to improving genomic predictions for *Fusarium* head blight resistance in wheat. Agronomy-Basel 10, 16 10.3390/agronomy10122008

[70] Manstretta V. and Rossi V. (2016) Effects of temperature and moisture on development of *Fusarium graminearum* perithecia in maize stalk residues. Appl. Environ. Microbiol. 82, 184–191 10.1128/AEM.02436-15 26475114 PMC4702647

[71] Rossi V., Giosuè S., Pattori E., Spanna F. and Del Vecchio A. (2003) A model estimating the risk of *Fusarium* head blight on wheat. EPPO Bulletin 33, 421–425 10.1111/j.1365-2338.2003.00667.x

[72] Zhang X., Li C.J., Xue B.Y., Ji P.S., Li Y.G., Sun L. et al. (2022) Development of a rapid sporulation method of *Fusarium graminearum* using liquid cultivation. Plant Dis. 106, 34–38 10.1094/PDIS-05-21-0911-SR 34282928

[73] Schmidt-Heydt M., Parra R., Geisen R. and Magan N. (2011) Modelling the relationship between environmental factors, transcriptional genes and deoxynivalenol mycotoxin production by strains of two *Fusarium* species. J. R. Soc. Interface 8, 117–126 10.1098/rsif.2010.0131 20462881 PMC3024818

[74] Ramirez M.L., Chulze S. and Magan N. (2006) Temperature and water activity effects on growth and temporal deoxynivalenol production by two Argentinean strains of *Fusarium graminearum* on irradiated wheat grain. Int. J. Food Microbiol. 106, 291–296 10.1016/j.ijfoodmicro.2005.09.004 16236377

[75] Mannaa M. and Kim K.D. (2017) Influence of temperature and water activity on deleterious fungi and mycotoxin production during grain storage. Mycobiology 45, 240–254 10.5941/MYCO.2017.45.4.240 29371792 PMC5780356

[76] Sanchis V. and Magan N. (2004) Environmental conditions affecting mycotoxins. Mycotoxins in food: detection and control. In Woodhead Publishing Series in Food Science, Technology and Nutrition, pp. 174–189, Woodhead Publishing, Cambridge, UK

[77] Pei P.G., Xiong K., Wang X.Y., Sun B.G., Zhao Z.Y., Zhang X. et al. (2022) Predictive growth kinetic parameters and modelled probabilities of deoxynivalenol production by *Fusarium graminearum* on wheat during simulated storing conditions. J. Appl. Microbiol. 133, 349–361 10.1111/jam.15557 35365897

[78] Miraglia M., Marvin H.J.P., Kleter G.A., Battilani P., Brera C., Coni E. et al. (2009) Climate change and food safety: an emerging issue with special focus on Europe. Food Chem. Toxicol. 47, 1009–1021 10.1016/j.fct.2009.02.005 19353812

[79] Vary Z., Mullins E., McElwain J.C. and Doohan F.M. (2015) The severity of wheat diseases increases when plants and pathogens are acclimatized to elevated carbon dioxide. Global Change Biol. 21, 2661–2669 10.1111/gcb.12899 25899718

[80] Magan N., Medina A. and Aldred D. (2011) Possible climate-change effects on mycotoxin contamination of food crops pre- and postharvest. Plant Pathol. 60, 150–163 10.1111/j.1365-3059.2010.02412.x

[81] Masson-Delmotte V., Zhai P., Pirani A., Connors S., Péan C., Berger S. et al. (2021) Contribution of working group I to the sixth assessment report of the intergovernmental panel on climate change. Climate Change 2021: *The Physical Science Basis* Switzerland

[82] Walsh M., Backlund P., Buja L., DeGaetano A., Melnick R., Prokopy L. et al. (2020) Climate indicators for agriculture, United States Department of Agriculture. https://naldc.nal.usda.gov/download/7201760/pdf

[83] Martinez M., Biganzoli F., Arata A., Dinolfo M.I., Rojas D., Cristos D. et al. (2022) Warm nights increase *Fusarium* Head Blight negative impact on barley and wheat grains. Agric. For. Meteorol. 318, 12 10.1016/j.agrformet.2022.108909

[84] Daou R., Assaf J.C. and El Khoury A. (2022) Aflatoxins in the era of climate change: the mediterranean experience. In Aflatoxins-Occurrence, Detection and Novel Detoxification Strategies(Assaf J.C., ed.), IntechOpen, London, UK 10.5772/intechopen.108534

